# Supplementary material for: Predicting candidate genes from phenotypes, functions and anatomical site of expression
Source: Bioinformatics. 2020 Oct 14;37(6):853–60. doi: 10.1093/bioinformatics/btaa879 (PMC8248315; doi:10.1093/bioinformatics/btaa879)
Supplement: btaa879_Supplementary_Data [file btaa879_supplementary_data.pdf]

Supplementary materials: Predicting candidate genes from phenotypes, functions, and anatomical site of expression

Jun Chen, Azza Althagafi, Robert Hoehndorf

Computer, Electrical & Mathematical Science and Engineering Division, Computational Bioscience Research Center (CBRC), King Abdullah University of Science and Technology, 4700 KAUST

1 Ranking model

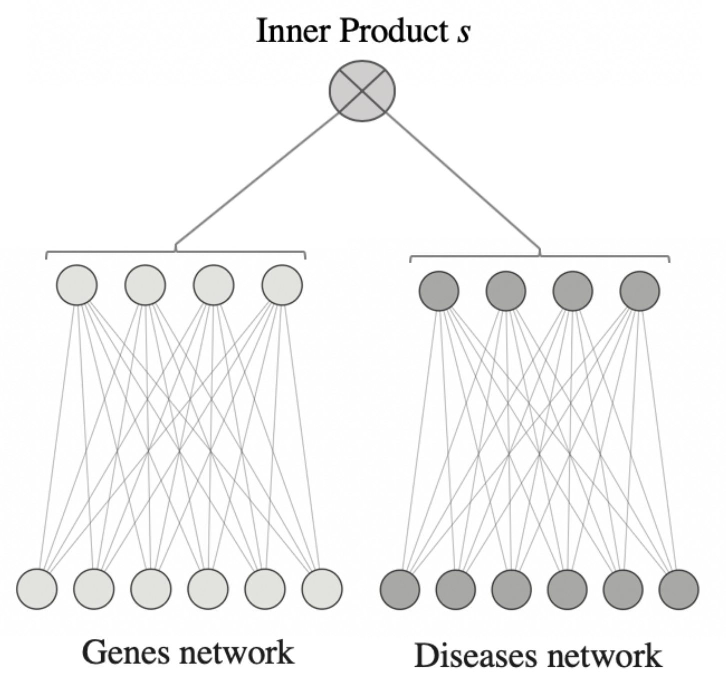

Figure 1: Ranking model with pointwise loss. We use this model to predict whether there should be a relation between the gene and disease (both of which are represented as "embeddings").

2 ROC curves for predicting gene–disease associations

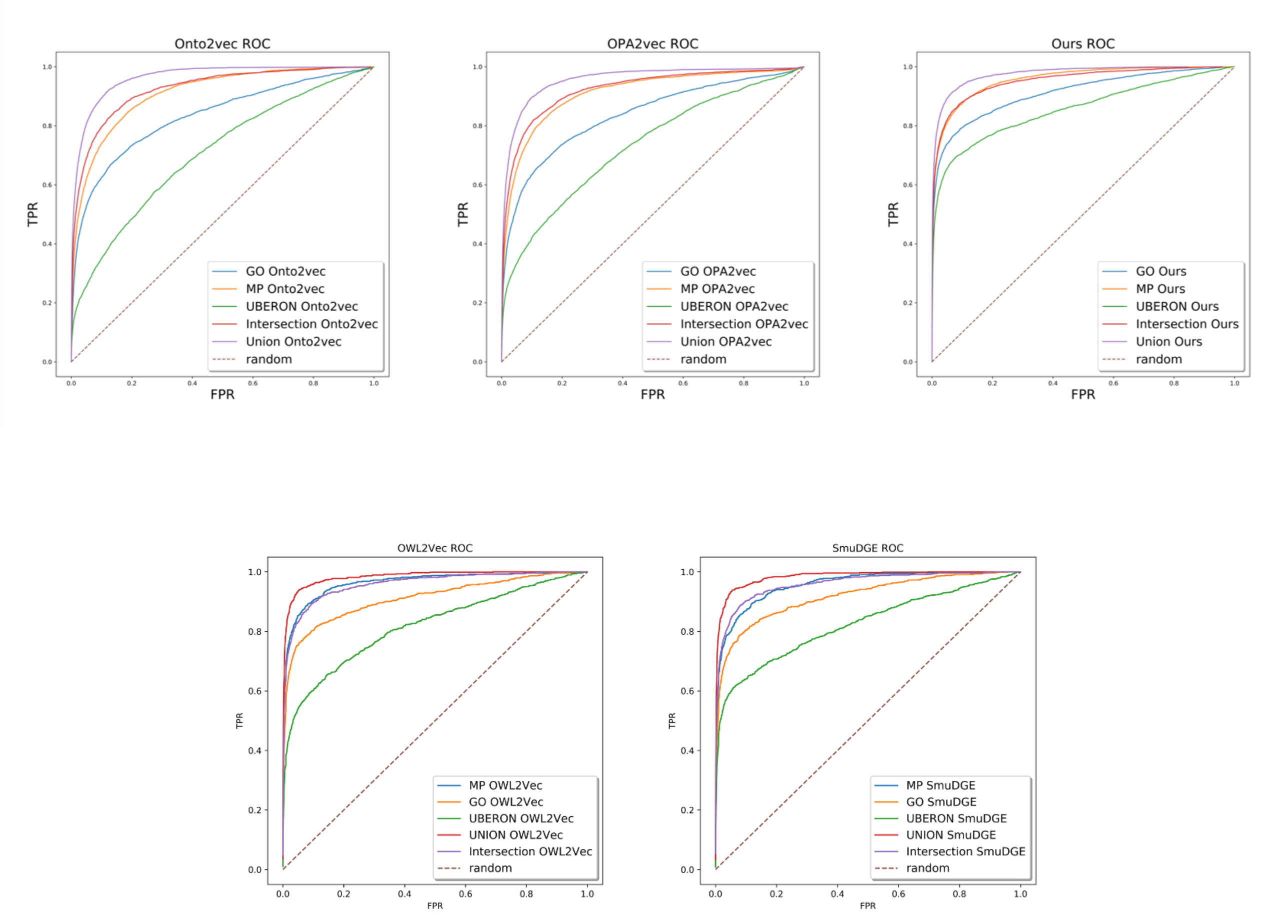

Figure 2: ROC curves for predicting gene–disease associations based on Onto2Vec, OPA2Vec, OWL2vec, SmuDGE and our method.

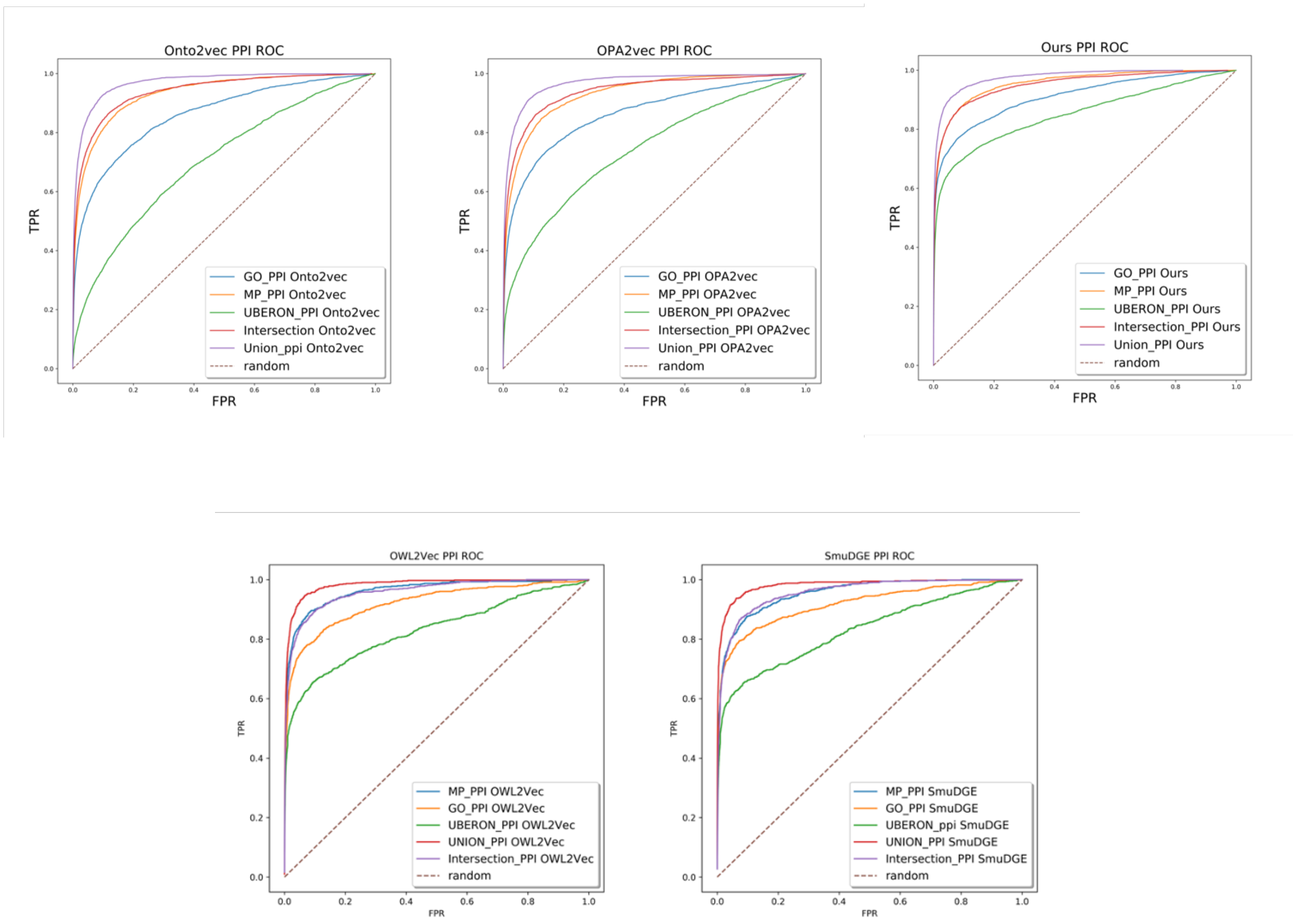

Figure 3: ROC curves for predicting gene–disease associations based on Onto2Vec, OPA2Vec, OWL2Vec, SmuDGE and our method when including interaction data.

3 Selection of gene expression thresholds

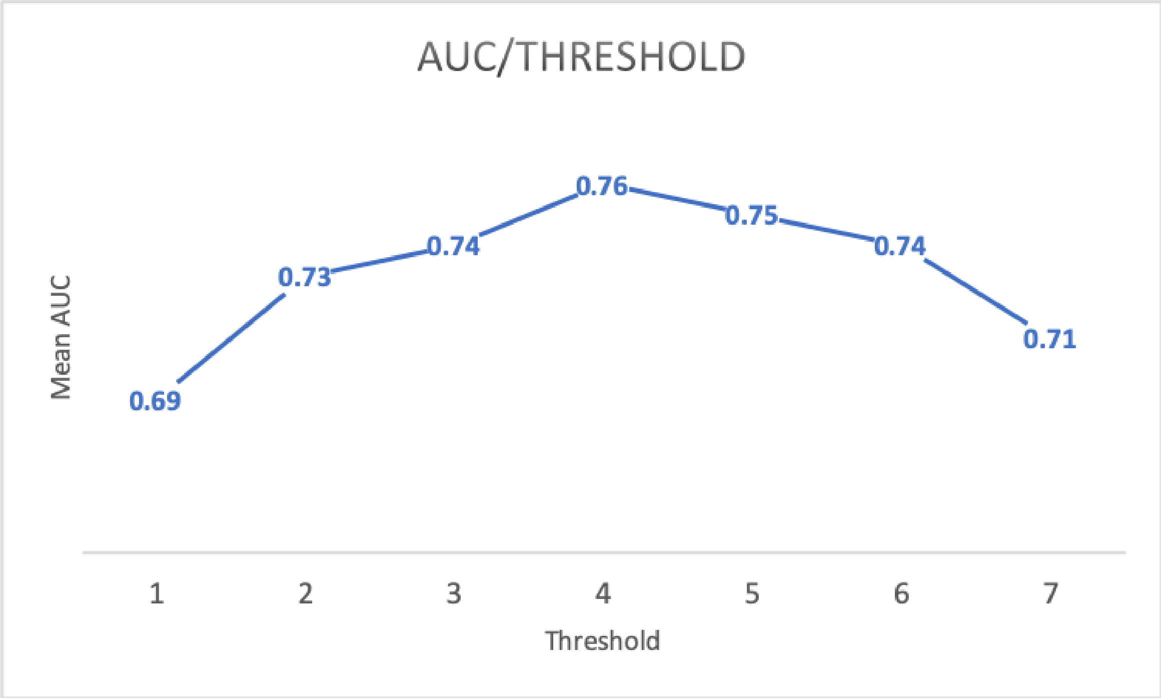

Figure 4: Onto2Vec AUC performance in 10-fold crossvalidation using different thresholds for gene expression in tissues.

Ontology	Genes	Diseases	Associations
MP	10,951	1,784	3,888
GO	17,786	1,784	3,883
UBERON	20,538	1,691	3,687
Intersection	9,886	1,687	3,655
Union	22,707	1,787	3,905

Table 1: Training and evaluation data used in our method. We list the annotations with each ontology, MP, GO, and UBERON, as well as the number of genes annotated with them and the number of diseases that these genes are associated with. *Intersection* represents the genes (and their associations) that have associations with all three ontologies, while *Union* represents the number of genes that have associations in one, two, or all ontologies.
